# Induction of CEMIP in Chondrocytes by Inflammatory Cytokines: Underlying Mechanisms and Potential Involvement in Osteoarthritis

**DOI:** 10.3390/ijms21093140

**Published:** 2020-04-29

**Authors:** Takashi Ohtsuki, Omer F. Hatipoglu, Keiichi Asano, Junko Inagaki, Keiichiro Nishida, Satoshi Hirohata

**Affiliations:** 1 Department of Medical Technology, Graduate School of Health Sciences, Okayama University, Okayama 700-8558, Japan; pebm12z8@cc.okayama-u.ac.jp (T.O.); farukjp@hotmail.com (O.F.H.); 2 Department of Molecular Biology and Biochemistry, Okayama University Graduate School of Medicine, Dentistry and Pharmaceutical Sciences, Okayama 700-8558, Japan; pazt4fck@s.okayama-u.ac.jp; 3 Department of Cell Chemistry, Okayama University Graduate School of Medicine, Dentistry and Pharmaceutical Sciences, Okayama 700-8558, Japan; jinagaki@cc.okayama-u.ac.jp; 4 Department of Orthopaediac Surgery, Okayama University Graduate School of Medicine, Dentistry and Pharmaceutical Sciences, 2-5-1,Shikata-cho, Kita-ku, Okayama 700-8558, Japan; knishida@md.okayama-u.ac.jp

**Keywords:** cell migration-inducing hyaluronidase 1 (CEMIP), chondrocyte, hyaluronan, mechanical strain, nuclear factor kappa B (NF-κB), osteoarthritis

## Abstract

In patients with osteoarthritis (OA), there is a decrease in both the concentration and molecular size of hyaluronan (HA) in the synovial fluid and cartilage. Cell migration-inducing hyaluronidase 1 (CEMIP), also known as hyaluronan (HA)-binding protein involved in HA depolymerization (HYBID), was recently reported as an HA depolymerization-related molecule expressed in the cartilage of patients with OA. However, the underlying mechanism of CEMIP regulation is not well understood. We found that CEMIP expression was transiently increased by interleukine-1β (IL-1β) stimulation in chondrocytic cells. We also observed that ERK activation and NF-κB nuclear translocation were involved in the induction of CEMIP by IL-1β. In addition, both administration of HA and mechanical strain attenuated the CEMIP induction in IL-1β-stimulated chondrocytes. In conclusion, we clarified the regulatory mechanism of CEMIP in chondrocytes by inflammatory cytokines and suggested the potential involvement in osteoarthritis development.

## 1. Introduction

The extracellular matrix of cartilage is composed mainly of hyaluronan (HA)–aggrecan (a major cartilage proteoglycan) network and type II collagens [1,2,3]. HA is a nonsulfated linear polysaccharide composed of N-acetylglucosamine and glucuronic acid [3]. HA is a major component of the synovial fluid (SF) and provides viscoelasticity retention, lubrication of joints, and cushioning against mechanical stress on articular cartilage [4]. In healthy subjects, hyaluronic acid in articular cartilage has a high molecular weight (HMW; 1200 kDa) and density (~2.5–4 mg/mL) [5]. HA assumes a helical configuration in a solution which can be attributed to hydrogen bonding between hydroxy groups along the chain [6]. As a result, HA can bind to and absorb water approximately 1000 times its own weight [7]. HMW HA-containing SF thus performs various functions such as cushioning, synovitis suppression, and elimination of reactive oxygen species [8]. On the other hand, due to degradation and/or depolymerization, the SF of osteoarthritis (OA) patients contains HA at a lower concentration (1–2 mg/mL) with a lower molecular weight (2–2.5 kDa) than the SF in healthy subjects [9]. Accordingly, HA degradation and/or depolymerization are closely related with the onset of OA. In a rat model of OA, injection of HA into the joint cavity protected the cartilage, suggesting an important role of HA in protecting the joint [10]. HA homeostasis is maintained through its synthesis and degradation and/or depolymerization [11]. However, the mechanism of HA depolymerization in OA is not fully understood. Although hyaluronidases (HYALs), such as HYAL2 and HYAL1, were long thought to be key enzymes for HA depolymerization [12], short interfering RNA (siRNA)-mediated knockdown of their gene expression failed to decrease HA-depolymerizing activity in skin fibroblasts and OA chondrocytes [13]. The cell migration-inducing hyaluronidase 1 (CEMIP), also known as hyaluronan (HA)-binding protein involved in HA depolymerization (HYBID; alias KIAA1199), is another molecule implicated in the degradation of HA in the skin and the OA cartilage [14]. Treatment of these cells with siRNA for CEMIP dramatically abolished HA degradation [13]. However, the mechanism underlying the regulation of CEMIP expression in arthritic cartilage remains unclear.

In this study, we examined the induction of CEMIP expression by inflammatory cytokines, regulation of CEMIP by HA and mechanical strain, and the underlying intracellular signaling mechanisms in chondrocytic cells.

## 2. Results

### 2.1. Immunolocalization of CEMIP in OA Articular Cartilage

**Figure 1:**
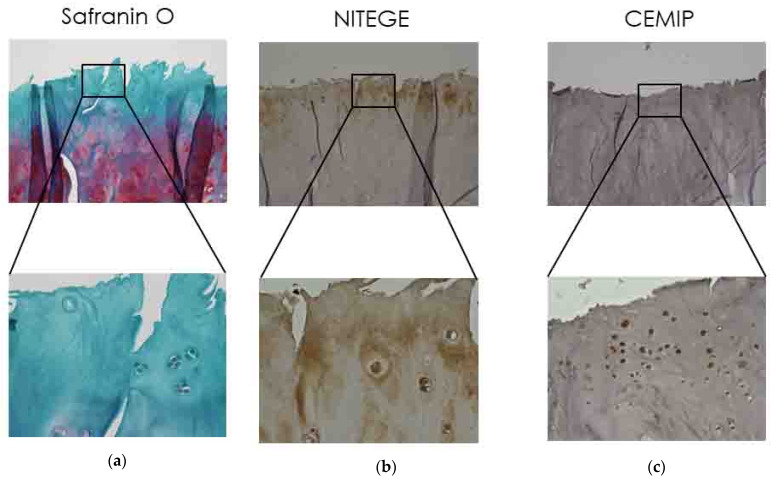
CEMIP expression was examined in OA cartilage. Serial sections of OA cartilage samples were subjected to Safranin O staining (**a**), immunostaining with antibodies to NITEGE (**b**), and CEMIP (**c**). Areas with severe OA show weak staining with Safranin O (**a**). Strong NITEGE-immunostaining was shown in damaged areas in OA cartilage (**b**). CEMIP-positive chondrocytes were located in the NITEGE-positive area of OA cartilage (**c**). The boxed areas in the top are shown at higher magnification at the bottom.

Serial sections of cartilage samples derived from patients with severe OA were subjected to histology with Safranin O staining and immunostaining with specific antibodies for CEMIP and NITEGE (aggrecan fragment cleaved by ADAMTS). In OA patients’ cartilage, the CEMIP expression was observed in transitional and radial zones (Figure 1). CEMIP-positive chondrocytes (Figure 1c) were located in the NITEGE-positive areas (dark brown, Figure 1b) of OA cartilage.

### 2.2. Effects of Inflammatory Cytokines on CEMIP mRNA Expression in Chondrocytic Cells

**Figure 2:**
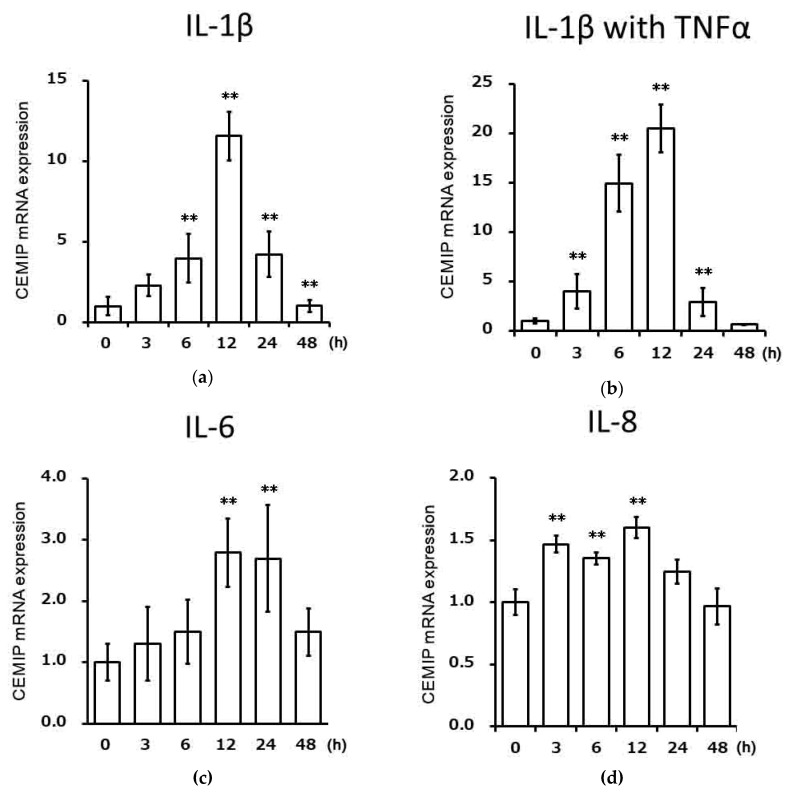
CEMIP mRNA induction by various cytokines. OUMS-27 cells were stimulated with cytokines for various durations (hours) as indicated. (**a**) Cells were treated with 10 ng/mL IL-1β. (**b**) Cells were treated with two cytokines, IL-1β and TNFα (each 10 ng/mL). (**c**) Cells were treated with IL-6 and soluble IL-6 receptor (each 50 ng/mL). (**d**) Cells were treated with IL-8 (100 ng/mL). Levels of CEMIP mRNA were measured relative to the levels of mRNA found in the unstimulated control cells. Values represent mean ± SD (*n* = 6 per group). ** *p* < 0.01 vs. control.

We examined the effects of various inflammatory cytokines on CEMIP mRNA expression in OUMS-27 cells using qRT-PCR. IL-1β significantly induced CEMIP mRNA expression starting at 6 h and reached its peak at 12 h (Figure 2a). CEMIP mRNA expression also increased in OUMS-27 cells stimulated by the combination of IL-1β and tumor necrosis factor (TNF)α (Figure 2b), compared to that with IL-1βalone. IL-6 together with soluble IL-6 receptor (Figure 2c) and IL-8 (Figure 2d) also increased the CEMIP mRNA expression.

### 2.3. IL-1β Induces CEMIP Protein Expression

**Figure 3:**
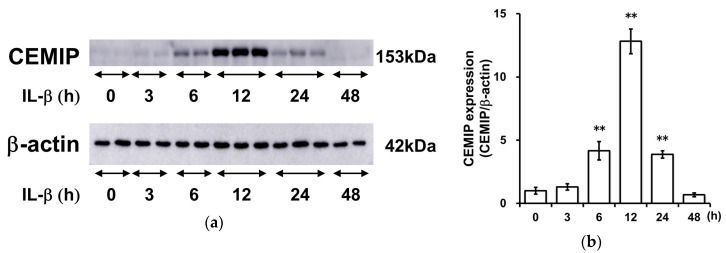
IL-1β induces CEMIP protein expression. (**a**) OUMS-27 cells were treated with IL-1β for 0 to 48 h. CEMIP protein was then detected by Western blot analysis. (**b**) Results of densitometric analysis. The densitometric values of immunoreactive bands for CEMIP were divided by respective values for β-actin. The normalized data are expressed as fold change relative to the values in unstimulated cells. Values represent mean ± SD (*n* = 3 per group). ** *p* < 0.01 vs. control.

We then performed Western blotting analysis to examine whether IL-1β induces CEMIP expression at the protein level. CEMIP protein expression was induced at 6 h and peaked at 12 h following IL-1β stimulation (Figure 3).

### 2.4. Signal Transduction Pathway Involved in CEMIP Induction

**Figure 4:**
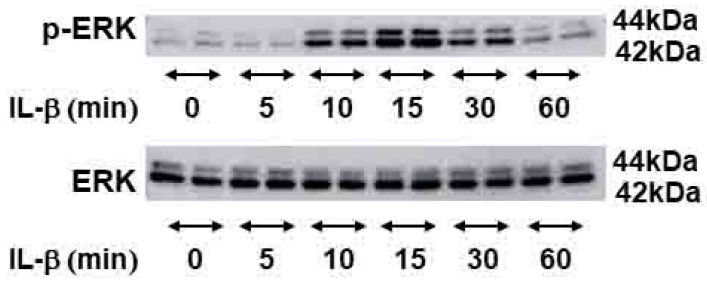
IL-1β induced phosphorylation of ERK in OUMS-27 cells. ERK and phosphor-ERK proteins were detected by Western blotting analysis. OUMS-27 cells were treated with IL-1β and subjected to Western blot at various time points (minutes) as indicated. Data shown are for experiments performed in duplicates.

**Figure 5:**
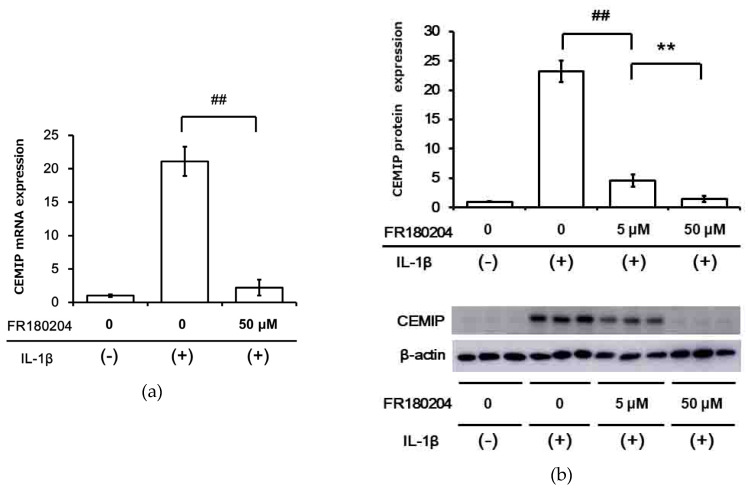
ERK inhibitor attenuated IL-1β-induced CEMIP expression in OUMS-27 cells. (**a**) ERK inhibitor (FR180204, 50 μM) was added 1 h prior to IL-1β stimulation and CEMIP mRNA expression was analyzed 12 h after treatment with IL-1β. (**b**) FR180204 attenuated IL-1β-induced CEMIP protein expression at 12 h in a dose-dependent manner. Values represent mean ± SD (*n* = 6 per group). ## *p* < 0.01 vs. IL-1β-treated group. ** *p* < 0.01 vs. IL-1β with low dose (5 μM) FR180204 treated group.

IL-1β has been reported to activate the ERK signaling pathway in other systems. Therefore, we examined ERK activation in OUMS-27 cells after stimulation with IL-1β. ERK phosphorylation was detected as early as 10 min after IL-1β stimulation, reached its peak at 15 min, and then gradually decreased until reaching a level similar to that observed in control cells after 60 min of IL-1β stimulation (Figure 4). Interestingly, pretreatment of OUMS-27 cells with FR180204 (an ERK inhibitor) significantly inhibited IL-1β-induced CEMIP mRNA expression (Figure 5a). We also confirmed that FR180204 attenuated CEMIP induction at the protein level in a dose-dependent manner (Figure 5b).

**Figure 6:**
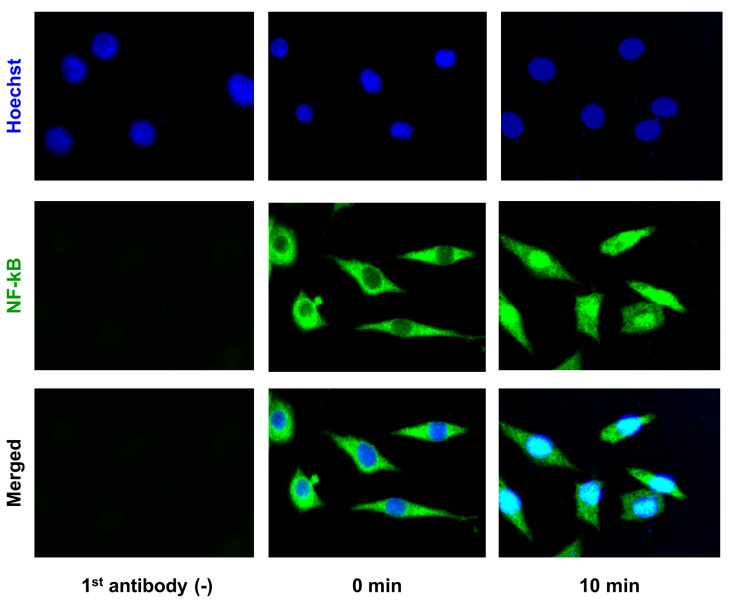
IL-1β induces translocation of NF-κB (p65) from cytoplasm to nucleus. OUMS-27 cells were seeded in collagen-coated chamber for 48 h. After 24 h of serum starvation, OUMS-27 cells were stimulated with IL-1β for 12 h. NF-κB p65 (green) was detected by immunocytochemistry (green) and nuclei were stained with Hoechst 33258 (blue). Merged images are shown at the bottom.

**Figure 7:**
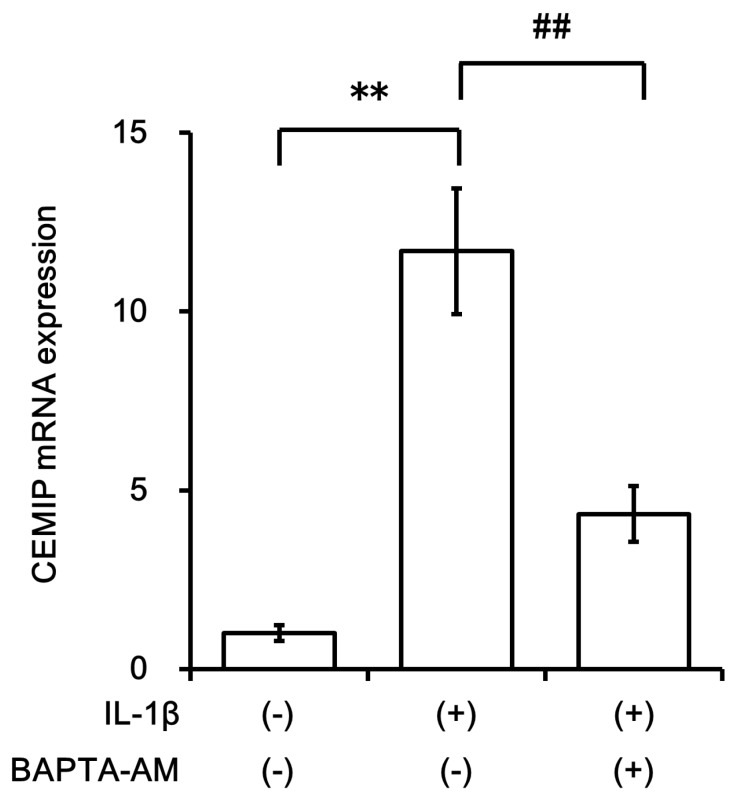
NF-κB inhibitor (BAPTA-AM) attenuated IL-1β-induced CEMIP expression. OUMS-27 cells were treated with IL-1β for 12 h with or without 30 μM BAPTA-AM. The CEMIP mRNA expression level was measured by qRT-PCR. Values represent mean ± SD (*n* = 6 per group). ** *p* < 0.01 vs. control. ## *p* < 0.01 vs. IL-1β with BAPTA-AM-treated group.

We then analyzed nuclear translocation of NF-κB, a key transcription factor involved in signal transduction of inflammatory cytokines, using immunocytochemistry. Rapid nuclear translocation of NF-κB was observed within 10 min of IL-1β stimulation (Figure 6). Interestingly, when BAPTA-AM (NF-κB inhibitor) was added, the induction of CEMIP mRNA expression was attenuated under IL-1β stimulation, indicating that NF-κB activation was required, at least in part, for CEMIP mRNA induction by IL-1β (Figure 7).

### 2.5. HA Inhibits Inflammatory Cytokine-Induced CEMIP at mRNA and Protein Levels

**Figure 8:**
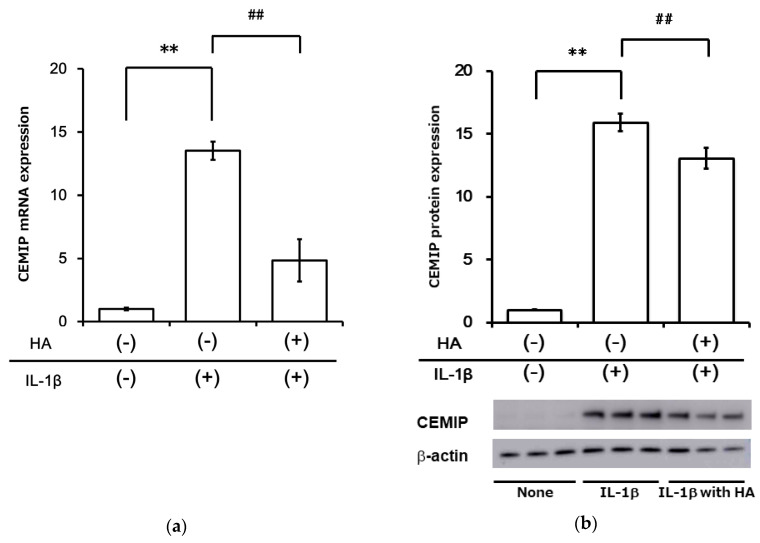
Hyaluronan preincubation attenuated inflammatory cytokine-induced CEMIP expression in OUMS-27 cells. OUMS-27 cells were incubated for 3 h with HA or medium alone and then treated with IL-1β for 12 h. (**a**) CEMIP mRNA expression was measured by qRT-PCR. (**b**) CEMIP protein expression was examined by Western blotting. Relative CEMIP expression change was measured by densitometric analysis. Values represent mean ± SD (a, mRNA, *n* = 6 per group; b, protein, *n* = 3 per group). ** *p* < 0.01 vs. control. ## *p* < 0.01 vs. HA preincubation group.

We examined the effect of HA on IL-1β-induced CEMIP expression. Pretreatment with HA significantly attenuated IL-1β-stimulated CEMIP mRNA expression in OUMS-27 cells (Figure 8a). Five hours of HA stimulation without IL-1β-stimulation did not alter CEMIP mRNA expression levels (Ohtsuki et al., unpublished data). We also confirmed inhibition of IL-1β-stimulated CEMIP expression at the protein level using Western blotting (Figure 8b).

### 2.6. Mechanical Strain (Cycle Tensile Strain) Attenuated Inflammatory Cytokine-Induced Expression of CEMIP

**Figure 9:**
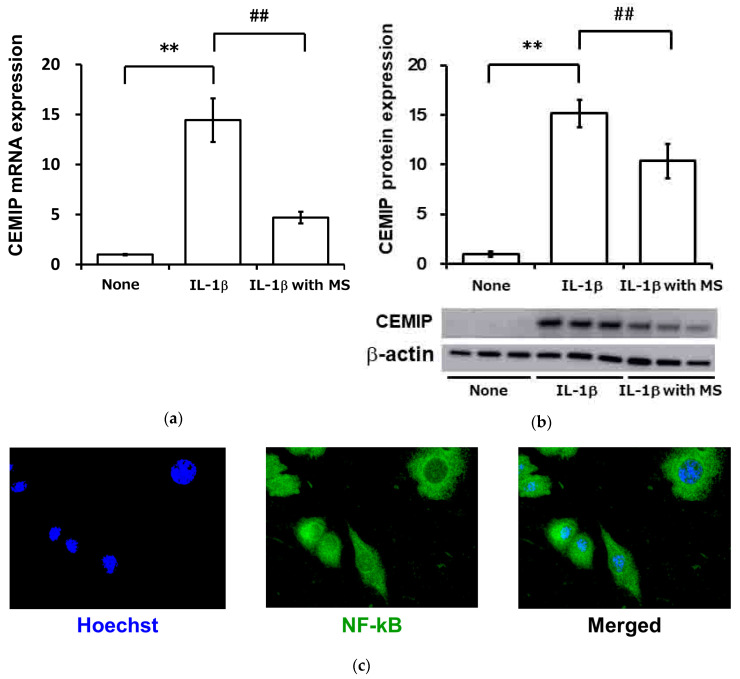
Mechanical strain attenuates IL-1β-induced CEMIP expression by inhibiting NF-κB (p65) translocation to the nucleus. OUMS-27 cells were treated with IL-1β and mechanical strain (0.5 Hz, 5% elongation) for 12 h. (**a**) CEMIP mRNA expression was then measured by qRT-PCR. (**b**) CEMIP protein expression was detected by Western blotting. Values represent mean ± SD (a, mRNA, *n* = 6 per group; b, protein, *n* = 3 per group). ** *p* < 0.01 vs. control. ## *p* < 0.01 vs. IL-1β-stimulated group with mechanical strain. (**c**) OUMS-27 cells were seeded on a collagen-coated stretch chamber. After 24 h serum starvation, the cells were treated with IL-1β for 10 min with or without MS (0.5 Hz, 5% elongation). NF-κB p65 (green) was detected by immunocytochemistry and nuclei were stained with Hoechst 33258 (blue).

We examined the effect of mechanical strain on IL-1β-induced CEMIP expression in OUMS-27 cells. Application of 5% cycle tensile strain at a frequency of 0.5 Hz resulted in the attenuation of CEMIP expression at both mRNA and protein levels (Figure 9a,b, respectively). IL-1β-induced NF-κB nuclear translocation was also strongly inhibited under mechanical strain (Figure 9c). NF-κB was retained in the cytoplasm under mechanical strain even after 6 h of IL-1β stimulation (Ohtsuki et al., unpublished data).

## 3. Discussion

In this study, we examined CEMIP expression in the cartilage derived from patients with OA and in cytokine-stimulated chondrocytic cells. We also elucidated the signaling mechanism involved in CEMIP induction by inflammatory cytokines and demonstrated that HMW HA and mechanical strain attenuated CEMIP induction in IL-1β-stimulated chondrocytic cells.

CEMIP has been studied in relation to various disorders. It was thought to play a central role in deafness, but the mechanism was unclear [15]. Upregulation of CEMIP has also been reported in multiple solid tumors, and it is recognized as an oncogenic protein. Moreover, CEMIP was reported to play a role in epithelial-to-mesenchymal transition (EMT) through Wnt/β-catenin signaling [16]. Yoshida et al. were the first to report a relationship between CEMIP and OA by showing that CEMIP is involved in HA catabolism [17]. Their group also reported that CEMIP plays a critical role in HA depolymerization independent of HYAL1 and HYAL2 in skin fibroblasts as well as in synovial fibroblasts [15]. HA metabolism is associated with OA development [18]. Previous studies have demonstrated that HA synthesis by HAS (hyaluronan synthase) was involved in OA development [19]. HYAL-1, HYAL-2, and CD44 were found to be increased in OA chondrocytes compared to normal cartilage, but their gene knockdown did not have a significant effect on HA degradation [20]. These data suggested that CEMIP might be associated with HA metabolism in OA cartilage. Therefore, we explored the mechanism of CEMIP regulation in chondrocytic cells. Shimizu et al. reported that CEMIP expression in OA chondrocytes was induced by TNFα, but not by TGF-β1, IL-1α, histamine, insulin-like growth factor (IGF)-1, vascular endothelial growth factor (VEGF)-165, basic fibroblast growth factor (bFGF), or prostaglandin E2 (PGE2) [20]. Interestingly, our results demonstrated that IL-1β induced CEMIP mRNA expression in OUMS-27 cells. Our results also showed that induction of CEMIP by IL-1β was an early and transient event lasting from 12 to 24 h after stimulation. Our results also suggested that the effects of IL-6 and IL-8 on CEMIP expression were rather negligible compared to the effect of IL-1β. Overall, we observed that CEMIP was one of the early response molecules expressed in chondrocytes after IL-1β stimulation.

High-affinity binding of IL-1β to its receptor (IL-1R) leads to activation of ERK in several cell types [21,22,23]. Our study also demonstrated that induction of ERK phosphorylation by IL-1β in OUMS-27 and a specific inhibitor of ERK blocked IL-1β-induced CEMIP expression in a dose-dependent manner. Although Akt phosphorylation was also induced by IL-1β, specific Akt inhibitors failed to attenuate CEMIP induction in OUMS-27 cells (Ohtsuki et al., unpublished data). Taken together, these data suggest that ERK activation is the main signaling pathway involved in CEMIP induction by IL-1β in chondrocytes.

Our group and others have previously reported that HMW HA has a protective role in inflammation [10,24,25,26]. We have also reported that HMW HA protects cartilage from aggrecan degradation in OUMS-27 as well as in rat OA models. HMW HA significantly attenuated the induction of mRNAs for ADAMTS4, ADAMTS9, and MMP-13 by IL-1β and TNFα. Intra-articular induction of HA also protected the cartilage from aggrecan degradation via inhibition of ADAMTS5 and ADAMTS9 mRNA expression in a rat model of surgical OA. We observed that aggrecan forms a complex with HA in the extracellular matrix (ECM). It should be noted that HA not only alters the expression of metalloproteinases (i.e., ADAMTSs and MMPs) induced by inflammatory cytokines, but also affects those of the ECM (i.e., aggrecan and collagens) downregulated by inflammatory cytokines (data not shown). These previous observations led us to hypothesize that HA regulates the induction of CEMIP in cytokine-stimulated cells. Results of the present study showed that treatment with HA in fact significantly attenuated CEMIP induction in IL-1β-stimulated cells.

The present study also revealed that NF-κB activation is involved in CEMIP induction in IL-1β-stimulated cells. The gene for CEMIP contains NF-κB binding sites in the promoter region [27]. It is well known that NF-κB is involved in the induction of matrix degradative molecules (ADAMTS4, 5, 9, 18, MMP-1, 2, 3, 8, 9, 13) following inflammatory stimulation in chondrocytes [28,29,30,31]. We have previously reported that HMW HA actually inhibited mRNA expression of ADAMTSs via inhibition of NF-κB activation, and it was independent of mechanical stress-induced runt-related transcription factor 2 (RUNX-2) nuclear translocation [29]. Soroosh et al. have reported that CEMIP was strongly expressed in fibroblasts derived from patients with Crohn’s disease, and HA fragments were produced that fostered inflammation and fibrosis [32].

It is known that mechanical strain has beneficial effects on cells and tissues [33,34]. We have recently found that mechanical strain effectively attenuated inflammatory cytokine-induced ADAMTS9 expression via transient receptor potential vanilloid type 1 (TRPV1) [35]. Our study demonstrated that mechanical strain dramatically inhibited IL-1β-induced NF-κB nuclear translocation and resulted in the attenuation of CEMIP expression both at the mRNA and protein levels. Further analyses confirmed that mechanical tensile strain inhibited IL-1β-induced nuclear translocation of NF-κB, indicating that mechanical tensile strain suppressed CEMIP transcripts by regulating NF-κB activation in this inflammation model.

There are several limitations to our study. First, to analyze the molecular mechanism underlying CEMIP induction in chondrocytes, we used OUMS-27 cells, and not chondrocytes from OA patients. Both normal chondrocytes (NHAC-kn) and OUMS-27 cells exhibit typical characteristics of chondrocytes, such as expression of the chondrocyte-specific ECM genes type II, IX, and XI collagen and aggrecan. Moreover, stimulation with inflammatory cytokine induced catabolic factors (ADAMTS4, 9, and MMP-13) with similar kinetics in both cell lines [35,36,37,38]. Furthermore, OUMS-27 cells and NHAC-kn responded to HA and MS in a similar manner [10]. Based on these results, we believed that OUMS-27 cells could serve as a model for investigating the molecular mechanism of CEMIP regulation. Second, we made no attempts to inhibit CEMIP or any upstream targets of CEMIP regardless of whether progression of OA was attenuated in the animal models. As such experiments are required in order to clarify the therapeutic potential of targeting CEMIP, we recommend that they be incorporated in future studies. Third, cell migration-inducing hyaluronidase 2 (CEMIP2), also known as transmembrane protein 2 (TMEM2), was also reported to be involved in HA depolymerization [39]. However, in a separate study, CEMIP2 expression in OA chondrocytes and OA synovial cells was not detected through qRT-PCR [13]. We found that CEMIP2 did not influence the initiation or development of OA. Nevertheless, the expression and functions of CEMIP2 in human OA joints have not been adequately studied. Finally, CEMIP expression was also observed in synovial cells. We, in fact, confirmed that CEMIP was induced in synovial cells by IL-1β but not by TNFα (data not shown). It is thought that synovial tissues including synovial cells are also stimulated by various external factors such as mechanical stress and inflammatory cytokines. The HA network in the synovial tissue may also be critical in relation to the role of CEMIP in human arthritis. These issues will be further examined in future studies.

It is plausible that there is a delicate balance between HA and HA-depolymerizing molecules (i.e., CEMIP). A slight degradation of HA may enhance the mRNA expression of CEMIP via IL-1β, which in turn leads to further degradation/depolymerization of HA. The precise underlying mechanism for this delicate interplay remains to be clarified. Nevertheless, intra-articular injection of HA is widely practiced for the treatment of patients with OA at clinics in Japan. The fact that exogenous administration of HA can not only enhance the net volume of HA but also attenuate its depolymerization by affecting CEMIP expression, as observed in this study, provides some mechanistic evidence in support of this clinical practice.

In conclusion, the results of this study demonstrated that inflammatory cytokines induced CEMIP expression in periarticular tissue. ERK phosphorylation and NF-κB translocation were involved in this cascade in chondrocytes. CEMIP might play an important role in the initiation or development of OA.

## 4. Materials and Methods

### 4.1. OA Cartilage Tissue Samples

This clinical study protocol conformed to the Declaration of Helsinki and was approved by the Okayama University Hospital ethics committee (Project code: 1803-018; Approved date: 1 June 2018). Written informed consent was obtained from all patients before the study. Tissues were collected from knee joints of OA patients following surgery and fixed with 4% paraformaldehyde, decalcified with 10% EDTA (pH 7.4), and embedded in paraffin.

### 4.2. Reagents

Recombinant human IL-1β, IL-6, soluble IL-6 receptor (sIL-6R), IL-8, and TNFα were purchased from R&D Systems (Minneapolis, MN, USA), stored at −80 °C, and diluted in culture medium immediately before use. ERK inhibitor (FR180204) was purchased from Sigma-Aldrich (St. Louis, MO, USA). Hyaluronan (2700 kDa) was kindly supplied by Chugai Pharmaceutical (Tokyo, Japan). Anti-KIAA1199 antibody (SAB2105467, Sigma-Aldrich) and anti-aggrecan neoepitope antibody (NITEGE) were purchased from Affinity BioReagents (Golden, CO, USA); anti-ERK (#4695, Cell Signaling Technology, Danvers, MA, USA), anti-phospho-ERK (#9101, Cell Signaling Technology, Danvers, MA, USA), anti-β-actin (A5441, Sigma-Aldrich), HRP-conjugated anti-rabbit IgG (55676, MP Biomedicals, Santa Ana, CA, USA), HRP-conjugated anti-mouse IgG (sc-2005, Santa Cruz, Dallas, TX, USA), anti-NF-κB p65 antibody (sc-372, Santa Cruz Biotechnology), Alexa 488-conjugated anti-rabbit IgG (Invitrogen, Carlsbad, CA, USA) were obtained as indicated in respective parenthesis.

### 4.3. Immunohistochemistry

CEMIP localization was assessed by immunohistochemistry in knee joint sections using anti-KIAA1199 antibody (1:1000) as previously described [10]. Deparaffinized sections were pretreated with chondroitinase ABC (1 U/mL; Sigma-Aldrich) at 37 °C for 2 h. The endogenous peroxidase was blocked with 3% H_2_O_2_ in PBS at 20 °C for 15 min and incubated in normal goat serum at 20 °C for 60 min. Rabbit anti-aggrecan neo antibody (10 mg/mL) was used as the primary antibody at 4 °C for 16 h. Histofine simple stain rat MAX PO(R) (Nichirei, Tokyo, Japan) was used as the secondary antibody [40]. The reaction was visualized by diaminobenzidine (DAB; Histofine simple stain DAB, Nichirei), resulting in a brown color. Counterstaining was carried out with hematoxylin. Sections incubated with normal rabbit non-immune serum or incubated without primary antibody were used as negative controls.

### 4.4. Cell Culture and Treatments

OUMS-27 chondrosarcoma cells were prepared as previously described [41,42]. The cells were cultured in Dulbecco’s modified Eagle’s medium (DMEM) containing 10% FBS and penicillin/streptomycin at 37 °C in a humidified atmosphere with 5% CO_2_ as previously described. 2.5 × 10^5^ cells were seeded in 6-well plates for 2 days, and the medium was replaced with serum-free medium for 24 h before cytokine stimulation, after which the cells were cultured in the presence of IL-1β and/or TNFα (10 ng/mL).

For studies related to intracellular signaling, cells were serum-starved and then preincubated for 1 h with specific inhibitors (for ERK, FR180204 (50 μM); for NF-κB, BAPTA-AM (30 μM)), before treatment with IL-1β (10 ng/mL) for 12 h.

For some experiments, cells were preincubated with HA (1 mg/mL) for 3 h, before treatment with IL-1β, as described above. Following further incubation for 12 h, the mRNA expression was determined by qRT-PCR and protein level was determined by Western blotting, as described below.

### 4.5. RNA Extraction and Real-Time Quantitative Reverse Transcription PCR (qRT)-PCR

Following cytokine stimulation, cells were washed twice with phosphate-buffered saline (PBS) and total RNA was extracted using TRIzol reagent (Invitrogen, Carlsbad, CA, USA), according to the manufacturer’s instructions, and reverse transcribed into cDNA as previously described [43,44]. Briefly, genomic DNA was removed by stimulation with 5 U DNase I (Roche Diagnostics, Lewes, UK) at 37 °C for 15 min, followed by enzyme inactivation at 65 °C for 10 min; 2 μg total RNA were reverse transcribed with random primers (Toyobo, Osaka, Japan). qRT-PCR was carried out on a StepOnePlus system (Applied Biosystems, Foster City, CA, USA) as previously reported, with slight modification [45,46]. Briefly, each reaction mixture contained 5 µL TaqMan Fast Advanced Master mix, 0.5 µL TaqMan Gene Expression assay for the target genes (*CEMIP*, *ADAMTS4, 9, MMP-13*) and the endogenous control (glyceraldehyde 3-phosphate dehydrogenase; *GAPDH*), and 4 µL cDNA. Cycling conditions were as follows: 95 °C for 20 s; and 40 cycles at 95 °C for 1 s and 60 °C for 20 s. All samples were analyzed in triplicate. TaqMan primers and probes (human *CEMIP*: assay ID Hs01552114_m1 based on Ref Seq NM_001293298.1; human ADAMTS4: assay ID Hs00192708_m1 based on RefSeq NM_005099.4; *ADAMTS9*: assay ID Hs00172025_m1 based on Ref Seq NM_182920.1; human MMP-13: assay ID Hs00233992_m1 based on RefSeq NM_002427.3; human GAPDH: assay ID Hs02758991_g1 based on Ref Seq NM_001256799.1) as well as the TaqMan Fast Advanced Master Mix were purchased from Applied Biosystems (Foster City, CA, USA). GAPDH was used to normalize the levels of target RNAs with the comparative Ct (ΔΔCT) method as previously described [47,48]. Values obtained from the untreated cells served as the control.

### 4.6. Protein Extraction, SDS-PAGE, and Western Blotting

Approximately 2.5 × 10^5^ OUMS-27 cells were independently seeded in 6-well plates and cultured for 48 h. Culture medium was replaced with FBS-free medium and then cultured for a further 24 h. Cells were lysed with CelLytic M Mammalian Cell Lysis/Extraction Reagent (Sigma-Aldrich) and centrifuged. Proteinase inhibitor (Roche, Basel, Switzerland) and phosphatase inhibitor cocktail (Sigma-Aldrich) were added to the lysis buffer to protect protein from degradation and phosphorylation. Protein concentration was measured using Pierce BCA Protein Assay kit (Thermo Fisher Scientific, Waltham, MA, USA), according to the manufacturer’s instruction. An amount of 10 μg OUMS-27 of cell lysates was used for Western blot analysis, as described previously [49,50]. The primary antibodies were anti-ERK (1:10,000), anti-phospho-ERK (1:10,000), and anti-β-actin (1:10,000). Secondary antibodies for anti-rabbit IgG (1:5000) and anti-mouse IgG (1:5000) were used and developed by using Amersham ECL Prime (GE Healthcare, Buckinghamshire, England, UK) [51]. All experiments were repeated at least three times, independently.

### 4.7. Evaluation of NF-κB Translocation

Cells (1.5 × 10^5^) were seeded in a stretch chamber coated with 0.1 mg/mL collagen and cultured for 2 days before being transferred to serum-free DMEM for 24 h as previously described [35]. After stimulation with IL-1β (10 ng/mL), the cells were treated with cold methanol for 30 min, followed by cold acetone for 10 min for permeabilization and fixation. After washing the cells in PBS, samples were blocked with 3% bovine serum albumin/PBS for 2 h, washed in PBS, and incubated overnight at 4 °C with anti-NF-κB p65 antibody (1:500). After PBS washes, cells were incubated for 1 h at 20 °C with Alexa 488-conjugated secondary antibody, then washed in PBS [52,53]. The nuclei were stained with Hoechst 33258 (1:5000) and the samples were mounted with coverslips and stored in the dark at 4 °C as previously described [54]. Images were obtained with a fluorescent microscope (BZ-X700; KEYENCE, Osaka, Japan).

### 4.8. Mechanical Strain

Cells (1.5 × 10^5^) were seeded in a stretch chamber coated with 0.1 mg/mL collagen and cultured for 2 days, then transferred to serum-free DMEM for 24 h [35,55]. The cells were then subjected to mechanical strain (cycle tensile strain) (5% elongation, 0.5 Hz) in the presence of IL-1β (10 ng/mL) using the ShellPa mechanical stretch system (Menicon Life Science, Aichi, Japan), which allowed for uniform stretching of the entire silicone membrane.

### 4.9. Statistical Analysis

Data are expressed as the mean ± S.D. For multiple comparisons, analysis of variance (ANOVA) was performed and post hoc analysis with Bonferroni’s test was employed.

## Abbreviations

ADAMTS	a disintegrin and metalloproteinase with thrombospondin motifs
BAPTA-AM	(1,2-bis(2-aminophenoxy) ethane-N,N,N′,N′-tetra acetic acid tetrakis (acetoxymethyl ester)
ECM	extracellular matrix
HA	hyaluronan
MMP	matrix metalloproteinase
NF-κB	nuclear factor κB
OA	osteoarthritis
